# Circulating tumour DNA (ctDNA) as a predictor of progression-free and overall survival in non-resectable pancreatic cancer: a systematic review and meta-analysis

**DOI:** 10.1016/j.jlb.2025.100441

**Published:** 2025-10-30

**Authors:** Mette M. Steiniche, Louise B. Callesen, Elizabeth H. Vlk, Lise Ventzel, Signe Timm, Rikke F. Andersen, Sidsel C. Lindgaard, Torben F. Hansen, Morten Ladekarl, Karen-Lise G. Spindler

**Affiliations:** a Department of Oncology, Aarhus University Hospital, Aarhus, Denmark; b Department of Clinical Medicine, Aarhus University, Aarhus, Denmark; c Department of Oncology, Regional Hospital Gødstrup, Denmark; d Department of Respiratory Medicine, Aarhus University Hospital, Aarhus, Denmark; e Department of Oncology, University Hospital of Southern Denmark, Vejle, Denmark; f Institute of Regional Health Research, Faculty of Health Sciences, University of Southern Denmark, Odense, Syddanmark, Denmark; g Department of Biochemistry and Immunology, University Hospital of Southern Denmark, Vejle, Denmark; h Department of Oncology, Copenhagen University Hospital - Herlev and Gentofte, Herlev, Denmark; i Department of Public Health, Section for Health Data Science & AI, Copenhagen University, Copenhagen, Denmark; j Department of Oncology and Clinical Cancer Research Center, Aalborg University Hospital, Aalborg, Denmark; k Department of Clinical Medicine, Aalborg University, Aalborg, Denmark

**Keywords:** Circulating tumor DNA (ctDNA), Cell-free DNA (cfDNA), ctDNA kinetics, ctDNA dynamics, Liquid biopsy, Response evaluation, RECIST / ctDNA-RECIST, Pancreatic ductal adenocarcinoma (PDAC), Pancreatic cancer, Unresectable pancreatic cancer, Advanced pancreatic cancer, Metastatic pancreatic cancer, Outcomes / kliniske endpoints, Treatment response, Prognosis, Survival, Overall survival (OS), Progression-free survival (PFS), Biomarkers, Predictive biomarker, Prognostic biomarker, Systematic review, Meta-analysis

## Abstract

**Background:**

This systematic review and meta-analysis synthesised current evidence on circulating tumour DNA (ctDNA) for predicting clinical outcomes in patients with non-resectable pancreatic ductal adenocarcinoma (PDAC).

**Methods:**

PubMed, Embase, and Cochrane databases were searched up to 31/01/2025. Eligible studies reported prognostic value of ctDNA in patients with non-resectable PDAC. Meta-analyses evaluated associations between baseline ctDNA and changes in ctDNA during treatment (ctDNA kinetics) and survival outcomes. Risk of bias was assessed using the Quality in Prognosis Studies (QUIPS) tool.

**Results:**

Sixty-four studies involving 5652 patients with non-resectable PDAC were included, with 24 studies contributing to meta-analyses. High baseline ctDNA level implied shorter overall survival (OS; HR = 2.3, 95 % CI 1.9–2.8; n = 1883) and progression-free survival (PFS; HR = 2.1, 95 % CI 1.8–2.4; n = 1196). Unfavourable ctDNA kinetics were associated with shorter OS (HR = 3.1, 95 % CI 2.3–4.3; n = 269) and PFS (HR = 4.3, 95 % CI 2.6–7.2; n = 244). Thirty-three studies had high risk of bias in at least one QUIPS domain.

**Conclusion:**

Baseline ctDNA and ctDNA kinetics demonstrate strong prognostic value in non-resectable PDAC. However, clinical translation is limited by methodological heterogeneity, notably the use of study-specific, non-validated thresholds. Standardised, externally validated thresholds for interpreting ctDNA changes are needed to support clinical implementation.

**Prospero:**

CRD42023438774.

## 1 Introduction

Pancreatic ductal adenocarcinoma (PDAC) is a highly lethal malignancy, with a 5-year survival rate of only 13 % [1]. More than 80 % of patients present with non-resectable disease, facing a median overall survival (OS) of under 1 year [2,3]. While combination chemotherapy improves OS, its benefits are often offset by toxicity, and many patients, already frail and malnourished, may not tolerate second-line treatment upon disease progression [[3], [4], [5], [6]]. This therapeutic challenge underscores the urgent need for reliable tools to evaluate treatment benefits in this patient population.

Current standard response evaluation relies on imaging, which has several limitations in PDAC. Objective response, as defined by Response Evaluation Criteria in Solid Tumors (RECIST), correlates only modestly with OS, and many patients have non-measurable disease, e.g., confined to the peritoneum or the primary tumour in the pancreas. In the latter case, the reproducibility and accuracy of measurements are usually compromised by the dense fibrotic stroma and peritumoral inflammatory changes [[7], [8], [9], [10]]. In addition, the treatment efficacy is first evaluated after 8–12 weeks of chemotherapy, potentially exposing patients to prolonged toxicity of an ineffective treatment. The only approved biomarker in PDAC, the tumour marker CA19–9, has inconsistent predictive value and is influenced by non-malignant conditions such as pancreatitis and cholestasis [[11], [12], [13]]. Moreover, 10–15 % of the population are non-expressors, which further reduces the clinical utility [[11], [12], [13]]. These limitations emphasise the need for more reliable and universally applicable biomarkers to inform treatment decisions.

Liquid biopsies have emerged as a promising approach to meeting this need. Circulating tumour DNA (ctDNA) in the blood has been studied for several years, and recent clinical studies have validated specific ctDNA assays for use across various tumour types [[14], [15], [16], [17]]. Being minimally invasive, rapidly measurable, and easily repeatable, ctDNA holds promise as a tool to optimize clinical management of patients with PDAC across various treatment settings. In the palliative context, its short half-life may allow earlier assessment of treatment benefits, enable timely treatment modifications, and support discontinuation of ineffective therapy [14,15], all of which are of particular importance in this patient population. However, while current ESMO guidelines recognise ctDNA as a promising tool for assessment of treatment efficacy, they do not endorse its routine use in PDAC [8,17].

This study presents a systematic review and meta-analysis evaluating the associations between ctDNA levels and kinetics and clinical outcomes, including response and survival, in patients with incurable, non-resectable PDAC.

## 2 Materials and methods

### 2.1 Eligibility criteria

Studies were eligible for inclusion if they met all the following criteria pre-specified in the review protocol: (1) studies involving patients with non-resectable PDAC; (2) studies reporting on the clinical value of ctDNA measured in blood plasma or serum at baseline and/or during systemic treatment; and (3) studies in which ctDNA was associated with OS, progression free survival (PFS) and/or treatment response. The review protocol was registered in PROSPERO (CRD42023438774) and follows the PRISMA reporting guidelines [18], checklist in Supplementary material 1. For criterion (1), studies including both non-resectable and resectable or borderline resectable disease were eligible if outcomes for the non-resectable subgroup were reported separately. For criterion (2), studies had to report the ctDNA levels or detectability at baseline, or changes in ctDNA levels or detectability during systemic treatment.

### 2.2 Search strategy

The databases PubMed, MEDLINE, EMBASE, and Cochrane Central Register of Controlled Trials were searched to identify relevant studies up to July 12th, 2023. Updated searches were conducted until January 31st, 2025. No restrictions were applied regarding language, study design, publication year, or publication type. All identified studies were screened for eligibility, but only studies with full-text data available were eligible for inclusion. The complete search strategy, including specific search strings, is presented in the Supplementary material 2**.**

### 2.3 Study screening and inclusion

Literature screening, data extraction, and quality assessment were conducted using the web-based software platform *Covidence* [19]. Each stage of the review process (screening, eligibility, inclusion, and data extraction) was independently performed by at least two researchers (MMS, LBC, EHV, LV). Following import from the selected databases, duplicates were automatically removed. Title and abstract screening was performed to categorise studies for inclusion, exclusion, or further assessment in cases of uncertainty. Full-text articles were then assessed within *Covidence*. Eligibility screening followed the predefined inclusion criteria, with disagreements resolved through discussion to reach consensus. Notably, no non-English studies were identified during screening.

Data extraction was conducted using a customised template to capture key study details, including first author, publication year, study design, number of patients, treatment (type and line), analytical method for ctDNA measurement, ctDNA marker, evaluated cut-off, treatment response, applied statistical test, and hazard ratios (HRs) for PFS and OS, along with 95 % confidence intervals (CIs), and p-values. Observational studies with prospectively collected data and samples for biomarker analysis were defined as prospective biomarker studies. Phase I/II trials tested investigational treatments and were classified as single-arm trials if lacking a comparator. Combined prospective biomarker study and randomized controlled trial (RCT) included biomarker discovery or validation sub-studies nested within RCTs, while exploratory studies focused on hypothesis generation.

### 2.4 Quality assessment

The risk of bias was evaluated across the following domains: study participation, study attrition, ctDNA measurement, outcome measurement, study confounding, and statistical analysis and reporting, following the Quality in Prognosis Studies (QUIPS) tool [20]. Three researchers (MMS, LBC, LV) independently assessed quality, and all papers were assessed by at least two researchers. Any discrepancies were resolved through discussion, involving a third party when necessary.

### 2.5 Synthesis of results

**Fig. 1:**
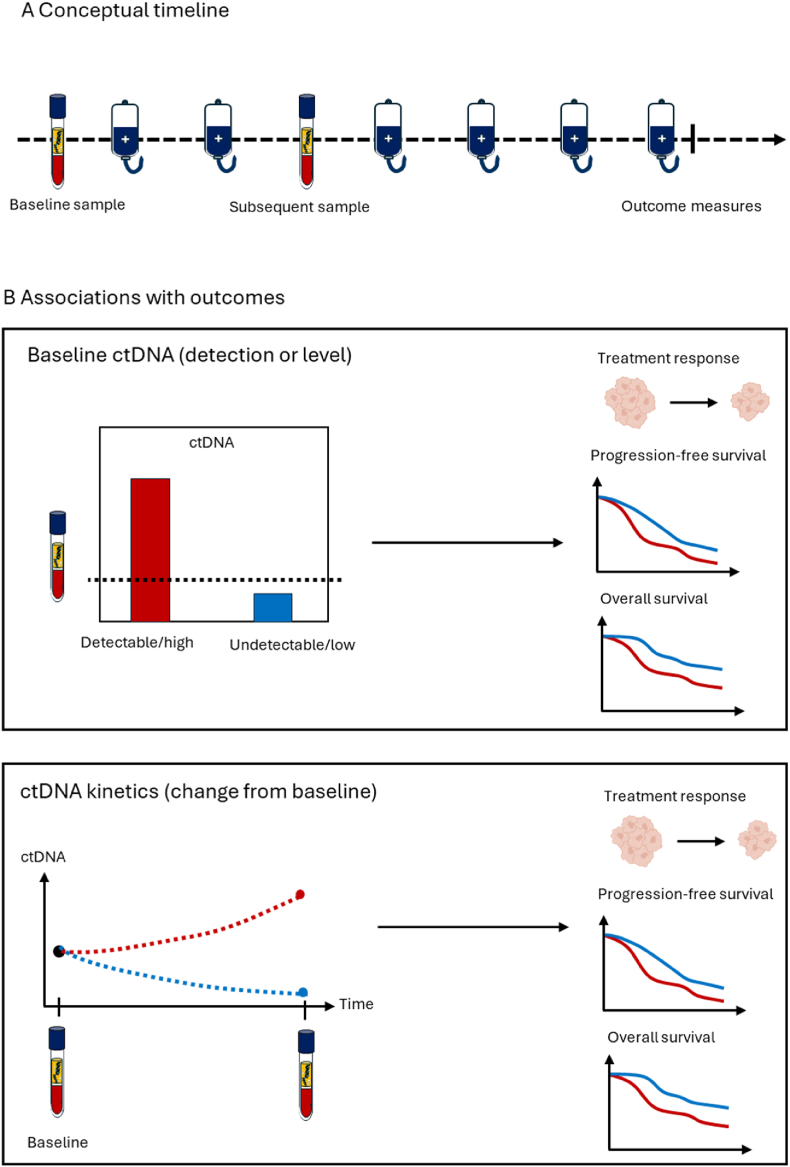
Conceptual illustration of ctDNA assessments and associations with clinical outcomes. Conceptual timeline illustrating sampling time points including baseline sample prior to initiation of the current treatment line and a subsequent sample later in the treatment course. In eligible studies, baseline ctDNA detection or level and/or change in ctDNA detection or level from baseline (ctDNA kinetics) were correlated to treatment response, progression-free survival and/or overall survival.

The clinical relevance of ctDNA as a prognostic biomarker in patients with non-resectable PDAC was evaluated based on two approaches: (1) baseline ctDNA levels or detection, and (2) on-treatment changes in ctDNA levels or detectability (referred to as ctDNA kinetics). Baseline ctDNA was defined as the level or detection of ctDNA before initiating systemic therapy. ctDNA kinetics referred to changes observed from baseline to a time point during treatment as reported in the individual studies (Fig. 1).

Core data were summarised in tables, including assessments of the association between baseline ctDNA or ctDNA kinetics and clinical outcomes. For meta-analyses, univariate HRs for PFS and OS were extracted as reported in the individual studies and presented in forest plots, with studies ordered chronologically by publication date. The HRs reflected comparisons based on study-specific thresholds for dichotomisation. Changes in ctDNA during treatment are referred to as favourable or unfavourable ctDNA kinetics.

In studies reporting multiple results for the same clinical outcome, for example, using different ctDNA markers or cut-offs, all results were included if they were derived from independent cohorts. If multiple results originated from the same cohort, pre-specified cut-offs were priorities; if not available, the analysis with the most complete data (HRs, 95 % CIs, p-value, and sample size) was included. In cases where data completeness was equal, the result covering the largest patient population across studies was prioritised. This selection principle was applied consistently across the meta-analyses. No quantitative synthesis was performed for treatment response outcomes due to limited reporting.

### 2.6 Statistical analysis

Studies providing HRs with corresponding 95 % CIs from univariate survival analyses were eligible for inclusion in meta-analyses estimating the association between ctDNA and clinical outcomes, specifically PFS and OS.

Clinical and methodological heterogeneity was assessed based on differences in study design, patient populations, blood sampling time points, ctDNA analytical method, ctDNA markers, and cut-off values. Study-specific results were visualised in forest plots.

Statistical heterogeneity was evaluated using visual inspection of forest plots and quantified using I^2^ statistics, with values above 50 % and 70 % considered indicative of moderate and high heterogeneity, respectively [21]. In line with the aim of summarising the existing literature, pooled estimates were reported unless the observed clinical or methodological heterogeneity exceeded expectations based on the pre-defined eligibility criteria. To account for the variability, a random-effects model was applied [22]. In parallel, a structured descriptive synthesis summarised findings from all included studies.

All analyses were conducted using Stata software version 18.5 (Stata Corp, College Station, TX, USA). A two-sided p-value <0.05 was considered statistically significant.

## 3 Results

### 3.1 Study selection and clinical context

**Fig. 2:**
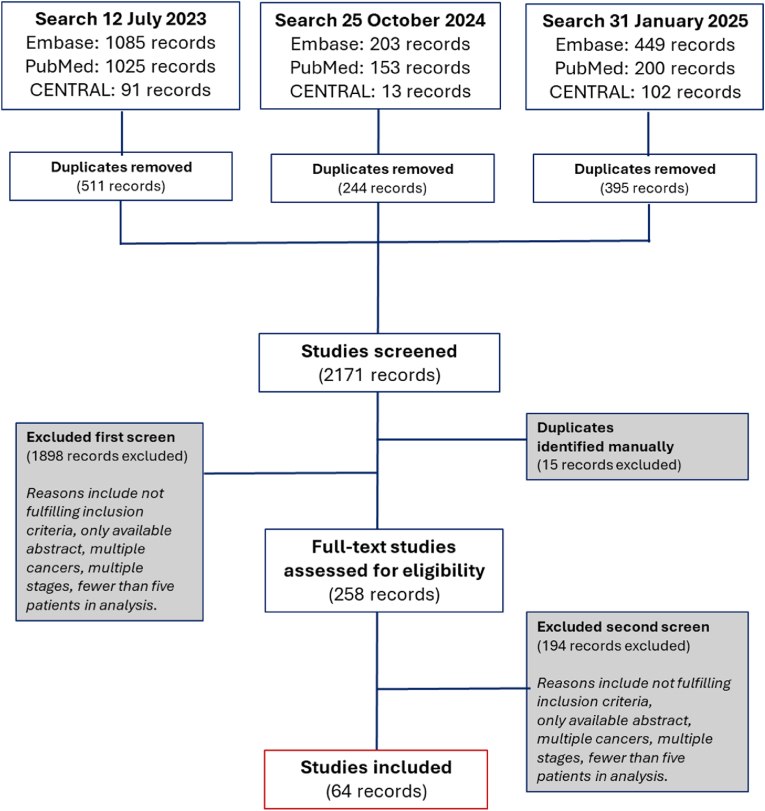
Flowchart of the study selection process. Overview of the identification, screening, eligibility assessment, and inclusion of studies.

A total of 2156 unique studies were identified and screened based on the eligibility criteria. Only studies with published full texts were included, resulting in 258 studies undergoing full-text review. The reasons for exclusion during this review included studies that reported on multiple cancer types, multiple disease stages, including resectable disease, no treatment or survival outcomes, used sample materials other than plasma or serum, or fewer than five in the analysis (Fig. 2). Ultimately, 64 studies met the inclusion criteria and were included in the final review (Fig. 2).

The studies included 6422 patients, 5652 of whom had non-resectable PDAC – 4814 patients (85 %) with metastatic disease (stage IV), and 838 (15 %) with locally advanced disease (stage III). Two studies did not specify the disease stages among patients with non-resectable PDAC. The association between ctDNA and treatment or survival outcomes was reported for at least 2073 patients receiving palliative systemic treatment of non-resectable PDAC. Of the included studies, 24 contributed to at least one meta-analysis.

Across studies, the most frequently reported treatments were gemcitabine-based, either alone or combined with nab-paclitaxel. FOLFIRINOX and other 5-FU-based therapies (e.g., FOLFOX, FOLFIRI, oral fluoropyrimidines) were also frequently used. Most studies focused on first-line treatment; only two included later-line therapies.

### 3.2 Baseline ctDNA and clinical outcomes

#### 3.2.1 Study characteristics

Sixty-three studies evaluated the prognostic impact of ctDNA detection or level before initiation of first- or later-line treatment (Table 1) [[23], [24], [25], [26], [27], [28], [29], [30], [31], [32], [33], [34], [35], [36], [37], [38], [39], [40], [41], [42], [43], [44], [45], [46], [47], [48], [49], [50], [51], [52], [53], [54], [55], [56], [57], [58], [59], [60], [61], [62], [63], [64], [65]]. These studies varied in study design, treatment line, analytical method, ctDNA marker, and ctDNA positivity threshold.

**Table 1:** Studies reporting on association between baseline ctDNA level or detection and treatment response and/or survival.

First author	Year	Study design	Line of treatment	Analytical method	PDAC (n)	Baseline ctDNA associated with			Reference number
						Treatment response	Progression free survival	Overall survival	
**Chen**	**2010**	**PBS**	**First-line**	**PCR amplification/direct sequencing**	**91**	**NA**	**NA**	**Yes**	39
Infante	2014	Prospective phase II trial	First-line	dPCR/BEAMing	160	NA	NA	D	82
Semrad	2014	Prospective phase II trial	First-line	ARMS PCR	30	NA	Yes	Yes	43
Tjensvoll	2015	PBS	First-line	qPCR	14	D	Yes	No	54
**Pietrasz**	**2016**	**PBS**	**First-line**	**ddPCR/targeted sequencing**	**135**	**D**	**NA**	**Yes**	74
Allenson	2017	PBS	Various/unknown	ddPCR	127	NA	NA	No	57
Cheng	2017	PBS	Various/unknown	ddPCR/targeted sequencing	211	D	NA	Mixed	64
Del Re	2017	PBS	First-line	ddPCR	27	No	No	No	40
Adamo	2017	PBS	First-line	Targeted sequencing	33	NA	NA	D	69
Henriksen	2017	PBS	First-line	Methylation specific PCR	95	NA	NA	Yes	38
**Chen**	**2017**	**PBS**	**First-line**	**Targeted sequencing**	**189**	**D**	**Yes**	**Yes**	42
Berger	2017	PBS	Various/unknown	Targeted sequencing	20	NA	NA	NA	86
Perets	2018	PBS	First-line	Targeted sequencing	17	NA	NA	Yes	72
**Park**	**2018**	**PBS**	**First-line**	**Deep sequencing/ddPCR**	**17**	**D**	**NA**	**NA**	60
Bernard	2018	PBS	First-line	ddPCR	194	Yes	D	Yes	78
Kim	2018	PBS	First-line	ddPCR	106	NA	No	No	51
Wei	2018	PBS	First-line	Targeted sequencing^a^	38	NA	NA	Yes	55
Kruger	2018	PBS	First-line	BEAMing	54	Yes	Yes	Yes	45
**Lapin**	**2018**	**PBS**	**First-line**	**Fragment length analysis**	**61**	**NA**	**Yes**	**Yes**	56
**Strijker**	**2019**	**PBS**	**First-line**	**NGS/targeted sequencing**	**60**	**D**	**NA**	**Yes**	58
Mohan	2019	PBS	First-line	ddPCR/targeted sequencing^a^	55	NA	NA	Yes	48
**Patel**	**2019**	**PBS**	**Various**	**Targeted sequencing**	**112**	**NA**	**NA**	**Yes**	79
**Sugimori**	**2019**	**PBS**	**First-line**	**dPCR**	**47**	**NA**	**No**	**NA**	70
**Watanabe**	**2019**	**PBS**	**First-line**	**ddPCR**	**78**	**NA**	**Yes**	**Yes**	50
Cheng	2020	PBS	First-line	ddPCR	210	NA	NA	Yes	53
**Bachet**	**2020**	**Phase** II RCT	**Second-line**	**Targeted sequencing**	**141**	**Yes**	**Yes**	**Yes**	85
**Toledano-Fonseca**	**2020**	**PBS**	**First-line**	**BEAMing**	**61**	**D**	**Yes**	**Yes**	66
Uesato	2020	PBS	Various/unknown	Targeted sequencing	104	D	Yes	Yes	73
Toledano-Fonseca	2021	PBS	First-line	BEAMing	58	NA	Yes	Yes	49
**Schlick**	**2021**	**PBS**	**Various/unknown**	**qPCR**	**42**	**NA**	**NA**	**Yes**	62
**Woo**	**2021**	**PBS**	**First-line**	**Whole genome sequencing**	**315**	**D**	**Yes**	**Yes**	61
**Stubbe**	**2021**	**PBS**	**First-line**	**Methylation specific PCR**	**100**	**NA**	**NA**	**Yes**	36
Pietrasz	2021	PBS/RCT	First-line	ddPCR/targeted sequencing	510	NA	Yes	Yes	35
Kirchweger	2021	PBS	Various/unknown	ddPCR	107	NA	NA	Mixed	68
**Botrus**	**2022**	**PBS**	**First-line**	**Targeted sequencing**	**104**	**Yes**	**Yes**	**Mixed**	81
Vrba	2022	PBS	Various/unknown	qPCR	19	NA	NA	Yes	75
Jo	2022	Single-arm phase I/II	First-line	Targeted sequencing	34	D	NA	NA	52
Huang	2022	PBS	First-line	Targeted sequencing^a^	74	D	NA	NA	77
**Chapin**	**2022**	**PBS**	**First-line**	**ddPCR**	**104**	**NA**	**NA**	**Yes**	63
Guan	2022	PBS	First-line	Targeted sequencing^a^	40	NA	Yes	Yes	76
**Renouf**	**2022**	**RCT (treatment arm)**	**First-line**	**Targeted sequencing**	**180**	**NA**	**Yes**	**Yes**	84
Kirchweger	2022	PBS	Various/unknown	ddPCR	70	Yes	Yes	Yes	65
Christenson	2022	PBS	First-line	Targeted sequencing	12	Yes	No	Mixed	80
**Aung**	**2022**	Phase II	**Second-line**	**Targeted sequencing**	**16**	**D**	**NA**	**NA**	67
Nitschke	2023	PBS	Various/unknown	ddPCR	108	NA	NA	Yes	44
Lapin	2023	PBS	First-line	Targeted sequencing	56	D	Yes	Yes	47
**Umemoto**	**2023**	**PBS**	**Various/unknown**	**Targeted sequencing**	**540**	**NA**	**NA**	**Yes**	71
Dayimu	2023	PBS/RCT	First-line	ddPCR	146	No	NA	Yes	83
Sellahewa	2023	PBS	First-line	ddPCR	81	NA	NA	Yes	34
Watanabe	2023	PBS	First-line	ddPCR	61	NA	NA	Yes	59
**Stubbe**	**2023**	**PBS**	**First-line**	**qPCR**	**96**	**NA**	**NA**	**Mixed**	37
**Evrard**	**2023**	**PBS**	**First-line**	**ddPCR**	**69**	**Yes**	**Yes**	**Yes**	46
Edland	2023	PBS	First-line	qPCR	81	Yes	Yes	Yes	41
Garcia-Ortiz	2023	PBS	First-line	ddPCR/BEAMing	44	NA	Yes	Yes	32
Shen	2023	PBS	Various/unknown	Targeted sequencing	153	D	NA	NA	33
Motobayashi	2024	PBS	First-line	ddPCR	20	NA	D	NA	31
Hu	2024	Prospective phase II	Various/unknown	Targeted sequencing	24	D	NA	NA	26
Huang	2024	PBS	First-line	Targeted sequencing^a^	43	NA	Yes	Mixed	29
**Koukaki**	**2024**	**PBS**	**First-line**	**qPCR**	**105**	**NA**	**NA**	**Mixed**	24
**Huerta**	**2024**	**PBS**	**First-line**	**ddPCR**	**80**	**NA**	**Yes**	**No**	25
Till	2024	PBS/phase **I/II**	First-line	ddPCR	214	NA	Yes	Yes	28
**Halkova**	**2024**	**PBS**	**First-line**	**PCR**	**118**	**NA**	**NA**	**Mixed**	27
**Hussung**	**2024**	**Exploratory study**	**First-line**	**ddPCR**	**47**	**D**	**Mixed**	**No**	23
**Kim**	**2025**	**PBS**	**First-line**	**Targeted sequencing**	**64**	**NA**	**No**	**No**	30

Abbreviations: PBS, prospective biomarker study, RCT, randomised controlled trial, PCR, polymerase chain reaction, dPCR, digital PCR, ddPCR, digital droplet PCR, NGS, next generation sequencing, qPCR, quantitative PCR, CGE, capillary gel electrophoresis, NA, not applicable, D, descriptive, PFS, progression-free survival, OS, overall survival. Studies eligible for meta-analysis marked with bold.

a Comprehensive genome profile assays (>300 genes).

#### 3.2.2 Study design and population

The studies were primarily designed as prospective biomarker studies (n = 52) ([24,25,27,[29], [30], [31], [32], [33], [34],[36], [37], [38], [39], [40], [41], [42]], [[44], [45], [46], [47], [48], [49], [50], [51],[53], [54], [55], [56], [57], [58], [59], [60], [61], [62], [63], [64], [65], [66]], [[68], [69], [70], [71], [72], [73], [74], [75], [76], [77], [78], [79], [80], [81]]), followed by phase II trials (n = 5) [26,43,67,82,85], a combination prospective biomarker study and randomised controlled trial (RCT) (n = 2) [28,35], single-arm trials (n = 2) [52,84], and exploratory studies (n = 2) [23,83]. The sample sizes ranged from 12 to 540 patients, with 38 studies (60 %) enrolling fewer than 100 patients ([23,25,26,[29], [30], [31], [32], [33], [34], [35], [36], [37], [38], [39], [40]], [[41], [42], [43], [44], [45], [46], [47], [48], [49], [50], [51], [52], [53], [54], [55], [56], [57], [58], [59], [60], [61], [62], [63], [64], [65], [66], [67], [68], [69], [70], [71], [72], [73], [74], [75], [76], [77], [78], [79], [80], [81], [82], [83]]).

In 49 studies, patients received first-line systemic treatment ([[23], [24], [25],[27], [28], [29], [30], [31], [32],[34], [35], [36], [37], [38], [39], [40], [41], [42], [43],[45], [46], [47], [48], [49], [50], [51], [52], [53], [54], [55], [56],[58], [59], [60], [61]], [63,66,69,70,72,74], [[76], [77], [78],[80], [81], [82], [83], [84]]). Only two studies reported on second-line treatment [67,85]. In the remaining studies, the treatment lines were either various or unknown [26,33,44,57,62,64,65,68,71,73,75,79].

#### 3.2.3 Analytical methods, ctDNA markers, and detection rates

For detection and quantification of ctDNA, most studies used either polymerase chain reaction (PCR) based analytical methods (n = 34) [[23], [24], [25],^28^,[30], [31], [32],^34^,[36], [37], [38], [39], [40], [41],[43], [44], [45], [46],^49^,^50^,^53^,^54^,^57^,^59^,^62^,^63^,^65^,^66^,^68^,^70^,^75^,^78^,^82^,^83^], targeted sequencing, e.g., Next Generation Sequencing (NGS) (n = 20) [26,29,30,33,42,47,52,55,58,67,[71], [72], [73],^76^,^77^,[79], [80], [81],^84^,^85^], or a combination of those (n = 6) [35,39,48,60,64,74]. Other approaches comprised capillary gel electrophoresis (n = 1) [27] and whole-genome sequencing (n = 1) [61].

A tumour-agnostic approach targeting KRAS mutations was the most widely used method of ctDNA detection and quantification (n = 36) [23,25,27,28,31,33,34,[39], [40], [41], [42], [43], [44], [45],[48], [49], [50], [51],^53^,^54^,^57^,^59^,^62^,^63^,[65], [66], [67], [68], [69], [70],^72^,^77^,^78^,[82], [83], [84]]. Five studies used small gene panels including up to eight genes (including KRAS) [46,55,58,64,80] Larger gene panels were used in 11 studies [26,30,52,71,73,74,76,77,79,81,85], and aberrant methylation patterns in one or more genes were used in 7 studies [24,32,[35], [36], [37], [38],^75^]. Of the five studies employing comprehensive genomic profiling assays [29,48,55,76,77], four focused on a limited gene subset [48,55,76,77], while only one reported results for the entire panel [29].

In 32 studies reporting specifically on patients with metastatic PDAC [[23], [24], [25], [26],^30^,^32^,^33^,^36^,^38^,[49], [50], [51],^57^,^58^,[64], [65], [66], [67], [68],[72], [73], [74], [75], [76], [77], [78], [79], [80], [81], [82], [83], [84], [85]], the baseline ctDNA detection rate ranged from 29 % to 100 %, with a 100 % detection rate observed in studies using large gene panels [29,76], KRAS-targeted sequencing [67] and methylation-specific PCR assays [75]. Studies presenting data from patients with locally advanced and metastatic PDAC combined (n = 25) [31,37,[39], [40], [41], [42], [43], [44], [45], [46], [47], [48],[52], [53], [54], [55], [56],^59^,^62^,^63^,^70^,^71^,^74^,^77^,^81^] found a ctDNA detection rate ranging from 33 % to 94 %. Only two studies reported the ctDNA detection rate specifically for locally advanced PDAC, with, respectively, 31 % and 74 % detection rates [57,81]. The remaining studies reported the detection rate across multiple disease stages or did not report it.

Most studies reported ctDNA status at baseline based on the assay's detection limit, either as the sole cut-off or as one of several reported thresholds (n = 51) ([[23], [24], [25]], [27,28,30,31,[33], [34], [35], [36], [37], [38], [39], [40], [41]], [43,45,48,[50], [51], [52], [53], [54], [55],^57^], [58,60,[62], [63], [64], [65]], [[67], [68], [69], [70], [71], [72], [73], [74], [75], [76],^78^,^79^], [[81], [82], [83], [84], [85]]). Six studies reported on a specific ctDNA concentration [42,44,46,49,59,66], and each of the remaining used a continuous ctDNA value, a genomic instability score, the median fragment length, the median mutant allele frequency, or only reported descriptive results [26,56,61,77,80].

#### 3.2.4 Overall survival

Thirty-six studies found a statistically significant association between baseline ctDNA above the chosen threshold and shorter OS [28,32,[34], [35], [36],^38^,^39^,[41], [42], [43], [44], [45],[47], [48], [49], [50],^53^,^55^,^56^,^58^,^59^,[61], [62], [63],^65^,^66^,[71], [72], [73],^75^,^76^,^78^,^79^,[83], [84], [85]], whereas six studies did not support this association [23,25,30,40,51,57]. In 10 studies reporting multiple analyses, results were diverging, depending on the disease stage, the chosen cut-off, or the ctDNA marker evaluated [24,27,37,46,54,64,68,77,80,81]. The remaining studies either presented baseline ctDNA in relation to OS descriptively without a statistical test or did not evaluate the association [26,31,33,52,60,67,69,70,77,82,86].

#### 3.2.5 Progression-free survival

In 17 studies, baseline ctDNA above the study-defined threshold implied a shorter PFS [25,28,32,35,41,47,49,50,61,65,66,73,77,78,81,84,85], whereas five studies did not find a statistically significant correlation [23,30,40,46,51,54,70,76,80]. Results were diverging in four studies, where multiple analyses within the same study led to varying associations with PFS(23,46,54,76). The remaining studies either presented the association without a statistical test or did not assess it [24,26,27,31,33,34,[36], [37], [38], [39],^44^,^48^,^52^,^53^,^55^,[57], [58], [59], [60],[62], [63], [64],[67], [68], [69],^71^,^72^,^74^,^75^,[77], [78], [79],^82^,^83^,^86^].

#### 3.2.6 Treatment response

Ten studies reported on treatment response assessed by imaging in relation to pre-treatment ctDNA status or level. Eight studies reported an association [41,45,46,65,78,80,81,85], while two failed to support it [40,83]. No common effect estimate was reported, precluding further quantitative analyses.

### 3.3 ctDNA kinetics and clinical outcomes

#### 3.3.1 Study characteristics

Forty of the included studies assessed the association between ctDNA kinetics during treatment and clinical outcomes (Table 2) [23,25,26,[28], [29], [30], [31], [32], [33],[40], [41], [42],[45], [46], [47],[50], [51], [52],^54^,^55^,[58], [59], [60], [61], [62],[64], [65], [66], [67],^70^,^72^,^74^,[76], [77], [78],^80^,^81^,^83^,^85^,^86^]. They were characterised by variation in sampling time points, analytical methods, ctDNA markers, and the cut-off for ctDNA kinetics.

**Table 2:** Studies reporting on association between ctDNA kinetics and treatment response and/or survival.

First author	Year	Study design	Cut-off dynamics	N	Second sample	ctDNA kinetics associated with			Reference
						Treatment response	Progression-free survival	Overall survival	number
Tjensvoll	2015	PBS	D	10	4 weeks	D	NA	NA	54
Pietrasz	2016	PBS	D	8	30–45 days	D	NA	NA	74
Cheng	2017	PBS	D	13	8 weeks	Yes	NA	NA	64
Del Re	2017	PBS	Increase vs. Stability/reduction	25	15 days	No	Yes	Yes	40
Chen	2017	PBS	D	32	Eval	D	NA	NA	42
Berger	2017	PBS	Increase vs. decline in MAF	20	4 weeks	NA	Yes	NA	86
Perets	2018	PBS	ctDNA slope	15	Var	NA	NA	Yes	72
Park	2018	PBS	D	8	Var	D	NA	NA	60
Bernard	2018	PBS	MAF>1 %	34	Var	Yes	NA	NA	78
Kim	2018	PBS	Increase	56	3 months	NA	No	No	30
Wei	2018	PBS	D	17	Eval	D	NA	NA	55
Kruger	2018	PBS	Any increase	NR	Serial	Yes	Yes	Yes	45
Strijker	2019	PBS	D	4	Var	D	NA	NA	58
**Sugimori**	**2019**	**PBS**	**Clearance vs. Persistence**	**21**	**4**–**8 weeks**	**NA**	**Yes**	**NA**	**70**
**Watanabe**	**2019**	**PBS**	**Emergence**	**39**	**3 months**	**NA**	**Yes**	**Yes**	**50**
Bachet	2020	Phase II RCT	Increase vs. decrease in VAF	71	28 days	Yes	Yes	Yes	85
Toledano-Fonseca	2020	PBS	*Var*	7	NR	D	NA	NA	66
Schlick	2021	PBS	KRAS Cq ratio	NR	1 month/Eval	D	NA	NA	62
Woo	2021	PBS	*Binary I-score groups*	18	2 months	D	NA	NA	61
Botrus	2022	PBS	Clearance	23	Serial	Yes	Yes	Yes	81
Jo	2022	Single-arm phase I/II	D	18	After C2	D	NA	NA	52
Huang	2022	PBS	D	NR	After C2	D	NA	NA	77
Guan	2022	PBS	D	24	Varying	D	NA	NA	76
**Kirchweger**	**2022**	**PBS**	**Reduction >57,9 % vs. <57,9 %**	**32**	**2 weeks**	**Yes**	**Yes**	**Yes**	**65**
Christenson	2022	PBS	Continuous KRAS	12	2 months	Yes	Mixed	Yes	80
Aung	2022	Phase II trial	D	10	C2D1, day 22	D	NA	NA	67
Lapin	2023	PBS	Reduction >10-fold vs. <10-fold	25	1–2 months	D	Mixed	Yes	47
**Dayimu**	**2023**	**Explorative biomarker in RCT**	**Any increase vs. Decrease**	**18**	**4 weeks**	**No**	**BA**	**Yes**	**83**
Watanabe	2023	PBS	molecularPD/SD vs. neg/CR	61	42 days	NA	NA	Yes	59
**Evrard**	**2023**	**PBS**	**KRAS detection**	**55**	**4 weeks**	**No**	**Yes**	**Yes**	**46**
Edland	2023	PBS	Var	59	4 weeks	D	NA	NA	41
Garcia-Ortiz	2023	PBS	Var	15	Serial	D	NA	NA	32
Shen	2023	PBS	Detection	12	NR	D	NA	NA	33
Motobayashi	2024	PBS	MAF increase	13	Serial	NA	D	NA	31
Hu	2024	Phase II trial	D	9	4–7 days	D	NA	NA	26
Huang	2024	PBS	Increase	31	Serial	D	NA	NA	29
**Huerta**	**2024**	**PBS**	**Reduction > 84,75 %**	**50**	**Eval**	**NA**	**Yes**	**Yes**	**25**
**Till**	**2024**	**Phase Ib/2 and PBS**	**Clearance**	**70**	**8 weeks**	**NA**	**Yes**	**Yes**	**28**
**Hussung**	**2024**	**Single-center expl**	**increase vs decrease/stable**	**25**	**55 days**	**D**	**Yes**	**Yes**	**23**
**Kim**	**2025**	**PBS**	**Clearance**	**53**	**Eval**	**NA**	**Yes**	**Yes**	**51**

Abbreviations: PBS, prospective biomarker study, RCT, randomised controlled trial, NR, nor reported, NA, not applicable, D, descriptive, PFS, progression-free survival, OS, overall survival. Studies eligible for meta-analysis marked with bold.

#### 3.3.2 Study design and population

A few studies were exploratory [23,83], four studies were phase II trials [26,28,67,85], one study was a single-arm phase I/II [52], whereas the majority of studies were prospective biomarker study [23,25,26,[28], [29], [30], [31], [32], [33],[40], [41], [42],[45], [46], [47],[50], [51], [52],^54^,^55^,[58], [59], [60], [61], [62],[64], [65], [66], [67],^70^,^72^,^74^,[76], [77], [78],^80^,^81^,^83^,^85^,^86^].

Thirty-two studies included patients receiving first-line systemic treatment [23,25,26,[28], [29], [30], [31], [32], [33],[40], [41], [42],[45], [46], [47],[50], [51], [52],^54^,^55^,[58], [59], [60], [61], [62],[64], [65], [66], [67],^70^,^72^,^74^,[76], [77], [78],^80^,^81^,^83^,^85^,^86^]. In two studies, patients were treated in the second line [67,85], while treatment lines were various or not reported in six studies [26,33,62,64,65,86].

In all but three studies [59,80,86], ctDNA kinetics were analysed for a subset of the included cohort. The sample size varied from 4 to 71 patients.

#### 3.3.3 Analytical methods and ctDNA markers

The analytical methods used for measuring changes in ctDNA largely mirrored those applied at baseline with PCR-based methods [23,25,28,31,32,40,41,46,50,51,54,59,62,65,66,70,78,83], and targeted sequencing [26,29,30,33,42,47,52,55,58,67,72,76,77,80,81,85,86] being the predominant methods. KRAS mutation was the most frequently targeted ctDNA marker in studies assessing ctDNA kinetics, either alone or in combination with a few other gene mutations [23,25,28,[30], [31], [32], [33],[40], [41], [42],[45], [46], [47],[49], [50], [51],^54^,^55^,^58^,^59^,^62^,[64], [65], [66], [67], [68],^70^,^72^,^76^,^78^,^80^,^81^,^83^,^86^], followed by multiple gene panels [26,29,52,60,74,85].

#### 3.3.4 Sampling timepoints and thresholds for ctDNA kinetics

In the analysis of ctDNA kinetics during systemic treatment, all studies evaluated a change in ctDNA in a blood sample collected at a time point during the treatment course (the subsequent sample) compared to the ctDNA in the baseline blood sample. The second sample was most frequently collected approximately 4 weeks after treatment start [41,46,47,54,62,83,85,86] but the time interval varied from 4 days [26] to 3 months [50,51] or the time of the first treatment response evaluation [25,30,42,55,80]. Several studies collected serial samples [26,29,31,32,45,65,81], while others analysed samples from various, unspecified time points [33,58,60,66,72,76,78].

In the further analysis of the association between ctDNA kinetics and clinical outcomes, studies dichotomised the populations differently. Nine studies compared patients with an increase versus a decrease/stability in the ctDNA level [23,29,31,40,45,51,83,85,86], four studies based on the change in ctDNA detectability [28,30,70,81], and three studies based on a data-driven cut-off for a decrease in the ctDNA level [25,47,65]. Three studies reported on various cut-offs [32,41,66], and a single study had pre-defined categories for molecular response based on change in ctDNA level [59]. Other presentations comprised a ctDNA slope [72], a mutant allele frequency above 1 % [78], a KRAS quantification cycle ratio [62], dichotomised instability score groups [61], KRAS as a continuous value [80], or descriptive presentations without statistical tests [26,42,52,54,55,58,60,64,67,74,76,77].

#### 3.3.5 Overall survival

Among 17 studies with a statistical assessment of the association between favourable ctDNA kinetics (e.g., clearance or decline) and longer OS, or unfavourable kinetics (e.g., increase, persistence or decline below a chose threshold) and shorter OS, 16 studies [23,25,28,30,40,45,46,50,56,59,65,72,80,81,83,85] found the association significant, while one study did not [51].

#### 3.3.6 Progression-free survival

In 13 studies, ctDNA kinetics were significantly associated with PFS [23,25,28,30,40,45,46,50,65,70,81,85,86]. In two studies analyses had diverging results depending on sampling interval [47] or ctDNA marker and cut-off [80], and one study did not find an association [51]. One study presented an association descriptively with no statistical test [31].

#### 3.3.7 Treatment response

The relationship between ctDNA kinetics and treatment response was evaluated using statistical analysis in 10 studies, of which seven found a significant association between ctDNA changes and response to treatment [45,64,65,78,80,81,85], while three did not [40,46,83]. There was no common effect estimate across studies, preventing comparative analyses. An additional 20 studies presented the relationship descriptively [23,26,29,32,33,41,42,47,52,54,55,58,[60], [61], [62],^66^,^67^,^74^,^76^,^77^].

### 3.4 Meta-analyses

**Fig. 3:**
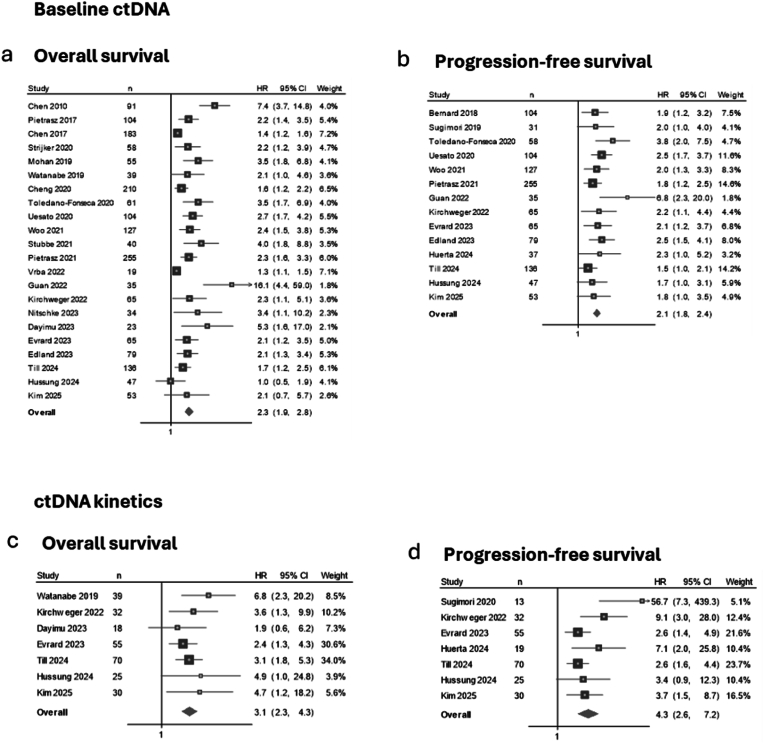
Associations between ctDNA and survival outcomes. Forest plots of the association between baseline ctDNA levels and overall survival (a) and progression-free survival (b), between ctDNA kinetics and overall survival (c) and progression-free survival (d) using the *random-effects model*. Heterogeneity assessed by I^2^ statistics was 75 % (a), 9 % (b), 0 % (c), and 50 % (d). Studies are presented in chronological order by publication date. *Baseline ctDNA* refers to the level or detection of ctDNA prior to initiation of systemic therapy. *ctDNA kinetics* refer to changes in ctDNA levels from baseline to a specific time point during treatment. The HRs reflect comparisons based on study-specific thresholds. ctDNA kinetics are denoted as favourable or unfavourable based on these thresholds. *Abbreviations*: n, number of patients included in the analysis; HR, hazard ratio; CI, confidence interval.

Baseline ctDNA levels above the specified thresholds were significantly associated with shorter OS in a meta-analysis of 22 studies including 1883 patients with advanced PDAC (pooled HR = 2.3, 95 % CI 1.9–2.8) (Fig. 3a). This analysis showed high statistical heterogeneity (I^2^ = 75 %). Similarly, higher/present baseline ctDNA was associated with shorter PFS in 14 studies (n = 1196), with a pooled HR of 2.1 (95 % CI 1.8–2.4) and low heterogeneity (I^2^ = 9 %) (Fig. 3b). For ctDNA kinetics, seven studies contributed data on OS, revealing minimal heterogeneity (I^2^ = 0 %). Unfavourable ctDNA kinetics, characterised by increases, persistence, or insufficient decline below the threshold, were associated with shorter OS (pooled HR = 3.1, 95 % CI 2.3–4.3, n = 269) (Fig. 3c). In the meta-analysis of ctDNA kinetics and PFS, also based on seven studies (n = 244), moderate heterogeneity was observed (I^2^ = 50 %), with unfavourable kinetics related to shorter PFS (pooled HR = 4.3, 95 % CI 2.6–7.2) (Fig. 3d).

### 3.5 Quality assessment

**Fig. 4:**
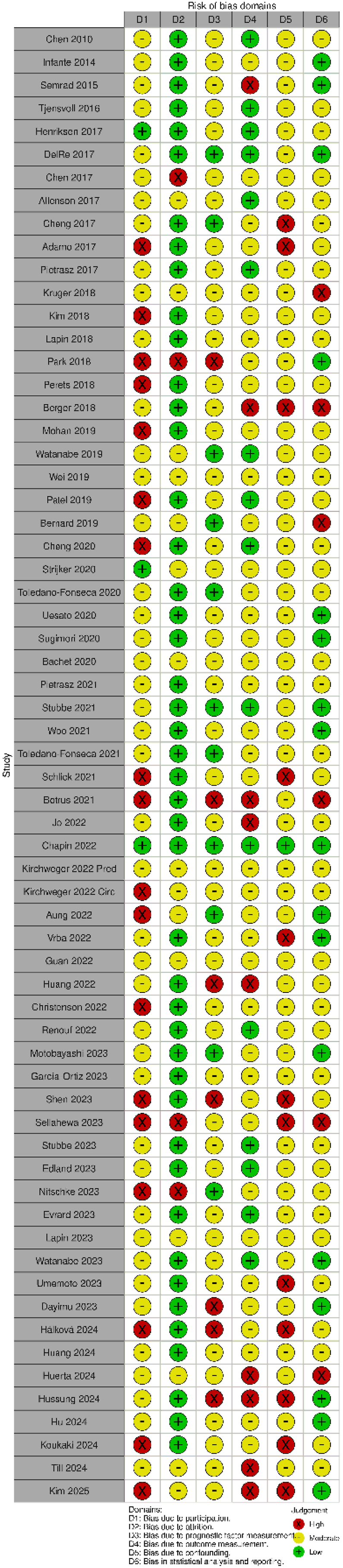
Quality assessment. Summary of risk of bias assessment assessed using the Quality in Prognosis Studies tool across included studies and domains. Each cell represents the risk of bias rating. Green indicates low risk, yellow indicates moderate risk, and red indicates high risk of bias.

Overall, the risk of bias across studies was rated moderate. Among the included studies, 33 were identified as having a high risk of bias in at least one domain. Study participation had the highest number of studies rated as high risk, with 18 studies falling into this category, followed by study confounding with 11 studies. In contrast, study attrition had an overweight of studies rated as low risk, with 47 studies in this category, while the other domains were predominantly rated as moderate. (Fig. 4).

## 4 Discussion

This systematic review and meta-analysis, comprising 5652 patients with non-resectable PDAC across 64 studies, found consistent associations between baseline ctDNA levels and poorer survival outcomes. Additionally, ctDNA clearance or decline during treatment was associated with improved OS and PFS. Despite the inter-study heterogeneity, the direction of the associations was remarkably consistent, supporting the potential of ctDNA as a prognostic biomarker in advanced PDAC.

The prognostic significance of baseline ctDNA aligns with findings from previous meta-analyses [[87], [88], [89], [90]]. However, the prognostic implications of ctDNA kinetics during palliative systemic treatment remain less explored.

Most studies included patients with metastatic disease, and only two studies reported outcomes separately for patients with locally advanced PDAC, limiting interpretation in a subgroup where radiological assessment can be particularly challenging [[7], [8], [9]]. In patients with metastatic PDAC, pre-treatment detection rates varied from 29 % to 100 %. Prior research found lower ctDNA levels in patients without distant metastases or with metastases confined to lungs, lymph nodes, or peritoneum compared to patients with liver metastases [71], underscoring the need to investigate biological variability. Low ctDNA levels may reflect limited shedding both in early-stage disease and in advanced PDAC without liver metastases [57,71,81]. This could be due to the typically desmoplastic nature of the primary tumor and represents a clinical challenge in this disease, placing high demands on the technical management of ctDNA analysis.

Additionally, methodological factors should be considered. Most studies used PCR-based techniques to analyse KRAS mutations, whereas targeted sequencing of broader gene panels, and more recently methylation-specific assays, reflect ongoing developments in ctDNA methods. With the highest detection rates found across different methods and targets, PCR-based tumour-agnostic approaches may be especially suitable for longitudinal monitoring of ctDNA in palliative treatment due to their feasibility, shorter turnaround times, and capacity to detect continuous changes.

KRAS mutations occur in more than 90 % of PDAC ([91]), making it an attractive target as a ctDNA biomarker. However, background noise in hotspot-targeted KRAS assays may compromise specificity. Increasing the threshold for a positive sample can limit false positives and improve reliability, but at the cost of sensitivity. Such a trade-off may contribute to the proportion of patients with ctDNA-negative samples, representing a central barrier to clinical utility. Further investigation of biological and methodological factors is needed, including assay refinement, error suppression techniques, and longitudinal ctDNA measurements.

In the present review, studies with low detection rates demonstrated associations with clinical outcomes, indicating that valuable prognostic information can be derived even in settings with limited detection. Given the wide variation in ctDNA detection rates, further investigation into the prognostic value of the ctDNA levels beyond dichotomised classifications is warranted. In addition, a comparison of ctDNA and CA19-9 at baseline and longitudinally is warranted to assess the value of either biomarker. However, this is challenged by the absence of well-defined thresholds for interpreting CA19-9 fluctuations during treatment.

Across the studies reporting on ctDNA kinetics, definitions of ctDNA response varied, with terms such as “clearance”, “decline,” and “undetectability” applied inconsistently. The timing of the second sample also ranged from a few days to several months after treatment initiation, adding to the methodological variability. In aggressive diseases such as PDAC, rapid clinical deterioration may preclude subsequent sampling, introducing a selection and immortal time bias. Nevertheless, the interstudy consistency of findings is notable. Future prospective studies with pre-defined early or longitudinal sampling and intention-to-treat analyses are warranted to confirm the associations.

Thresholds for ctDNA kinetics varied widely, were mainly data-dependent, and lacked external validation. Despite this variation, forest plots were visually consistent with similar effect sizes. This may indicate that the observed associations are robust despite methodological differences. However, the lack of standardised and validated criteria for ctDNA response remains a central barrier to clinical translation. Recently proposed predefined criteria, such as the ctDNA Response Evaluation Criteria in Solid Tumors (ctDNA-RECIST) [92], could provide a basis for standardization and should be prospectively validated in future studies.

Evidence from this review suggests an association between ctDNA kinetics and radiological response. However, the limited data constrain interpretability. Understanding the relationship between ctDNA kinetics and imaging may reveal insights into metastatic patterns and disease biology. Well-designed prospective studies with synchronised ctDNA and imaging assessments are needed to evaluate concordance and determine the distinct or complementary value of ctDNA.

The included studies were heterogeneous in design, methodology, and sample size. Most were retrospective, exploratory, and based on single-centre data. Most patients received standard first-line systemic treatment, which supports comparability across studies but limits insights into the value of ctDNA in later lines. ctDNA kinetics were often only reported for a subset of patients, reducing statistical power and potentially contributing to variability in the observed results. These selective analyses introduce a risk of immortal time bias, potentially affecting the estimated impact of ctDNA kinetics.

Another limitation is the moderate risk of bias across included studies. Notably, 33 studies had a high risk of bias in at least one domain, mainly concerning study participation and insufficient control of confounding. This could influence ctDNA levels and outcomes, limiting generalisability and introducing uncertainty into effect estimates. Consequently, results should be interpreted with that consideration in mind. In contrast, attrition bias was generally low, possibly reflecting the retrospective design of many studies, including patients with available ctDNA and outcome data.

The prognostic association between baseline ctDNA and survival was consistent with previous findings in other meta-analyses of both resectable and advanced PDAC [87], [88], [89], [90]. However, the meta-analysis of the association between baseline ctDNA and OS had the highest statistical heterogeneity (I^2^ = 75 %), indicating a high degree of variability in results. In addition, a proportion of studies were excluded from the analysis, introducing a risk of selection bias. This warrants cautious interpretation of the estimated effect size. The results are supported by the narrative synthesis with an in-depth description of the clinical and methodological variation, and the relation to survival outcomes across all studies.

In contrast, the meta-analyses of ctDNA kinetics showed low to moderate statistical heterogeneity (I^2^ = 0 % for OS and ctDNA kinetics; I^2^ = 50 % for PFS and ctDNA kinetics), which suggests a consistent association across the varying clinical and methodological characteristics of the included studies.

However, the selection bias inherent in retrospective studies may systematically exclude patients with poor performance status and prognosis, thereby limiting the generalisability to this group. Thus, findings may not be representative of all clinical scenarios [21] but do demonstrate the presence and direction of association among included patients.

As expected in a dataset dominated by retrospective analyses and exploratory approaches, many sources of heterogeneity contributed to a highly diverse overview. Current guidelines advocate for cautious interpretation of pooled estimates, supplemented by descriptive or narrative reporting to ensure clarity and transparency. Here, the objective was to summarise the existing literature, with less strict caution warranted than if the aim is to inform clinical decisions [93]. Thus, we judiciously presented pooled estimates, as they provide valuable insights to guide future research, despite the heterogeneity of the data.

This review highlights the prognostic relevance of baseline ctDNA and ctDNA kinetics in advanced PDAC, supporting its potential as a minimally invasive, early-response biomarker. Several studies demonstrate significant associations with OS, in some cases within two weeks of treatment initiation, indicating that ctDNA may offer earlier insight into treatment benefit than imaging.

While these findings support the clinical validity of ctDNA, prospective studies using standardised criteria and robust methodology are needed to establish clinical utility, specifically, whether ctDNA-guided treatment decisions improve outcomes over current standards [94].

Key areas for future research include underexplored populations such as patients with locally advanced PDAC, assay optimisation, the significance of ctDNA-negative status, and the predictive value of baseline levels for ctDNA response. A crucial next step will be the prospective validation of pre-defined thresholds, such as those proposed in ctDNA-RECIST [92], as well as the identification of optimal sampling time points and clinically relevant cut-offs. This will pave the way for assessing the real-world utility of ctDNA, including the impact on survival and quality of life of early treatment adaptation.

## Author contributions

MMS contributed to protocol writing, literature search, screening, data extraction, risk of bias assessment, manuscript drafting, and preparation of tables and figures. LBC supervised protocol writing and literature search, contributed to screening, data extraction, bias assessment, conducted statistical analyses, prepared figures, and reviewed the manuscript. EHV contributed to protocol writing, literature search, screening, figure preparation, and manuscript review. LV contributed to data extraction, bias assessment, and manuscript review. ST provided expertise on data presentation and statistical analyses and reviewed the manuscript. RFA provided expertise on molecular methodology and reviewed the manuscript. TFH, ML, and SCL advised on protocol development and reviewed the manuscript. KLS was responsible for conceptualisation, protocol supervision, overall project oversight and supervision, and manuscript review.

All authors contributed to the conception or interpretation of the work, drafted or revised the manuscript, approved the final version, and agree to be accountable for all aspects of the work.

## Ethical approval and consent to participate

This article is a systematic review and meta-analysis based solely on previously published studies. No new data were collected from human participants or animals. Therefore, ethical approval and informed consent were not applicable.

## Declaration of generative AI and AI-assisted technologies in the manuscript preparation process

During the preparation of this work the author(s) used ChatGPT (OpenAI) in order to improve the language of the manuscript. After using this tool/service, the author(s) reviewed and edited the content as needed and take(s) full responsibility for the content of the published article.

## Funding

This work was supported by grants from the Health Research Foundation of Central Denmark Region (A4796, A3656), The Novo Nordisk Foundation (NNF23OC0085224) and the Danish Cancer Society (R343-A19765). The funders had no role in the design or conduct of the study.

## Declaration of competing interest

The authors declare the following financial interests/personal relationships which may be considered as potential competing interests:Mette M. Steiniche reports financial support was provided by The Health Research Foundation of the Central Denmark Region. Karen-Lise Garm Spindler reports financial support was provided by The Health Research Foundation of the Central Denmark Region. Karen-Lise Garm Spindler reports financial support was provided by The 10.13039/501100009708Novo Nordisk Foundation. Karen-Lise Garm Spindler reports financial support was provided by 10.13039/100008363The Danish Cancer Society. If there are other authors, they declare that they have no known competing financial interests or personal relationships that could have appeared to influence the work reported in this paper.

## Data Availability

The full texts of included studies were retrieved from PubMed, Embase, the Cochrane Database of Systematic Reviews, and the Cochrane Central Register of Controlled Trials. Data used in this systematic review and meta-analysis are public and available from the online databases. Data collection templates, extracted data, analysis datasets, and analytical code used in this study are available from the corresponding author upon request.
